# Supplementary material for: Genetic variation in human drug-related genes
Source: Genome Med. 2017 Dec 22;9:117. doi: 10.1186/s13073-017-0502-5 (PMC5740940; doi:10.1186/s13073-017-0502-5)
Supplement: Supplementary file 1 — All supplemental figures cited in the text. Details about each figure are provided in the figure legends below the figures. (PDF 2264 kb) [file 13073_2017_502_MOESM1_ESM.pdf]

Supplementary Figure 1

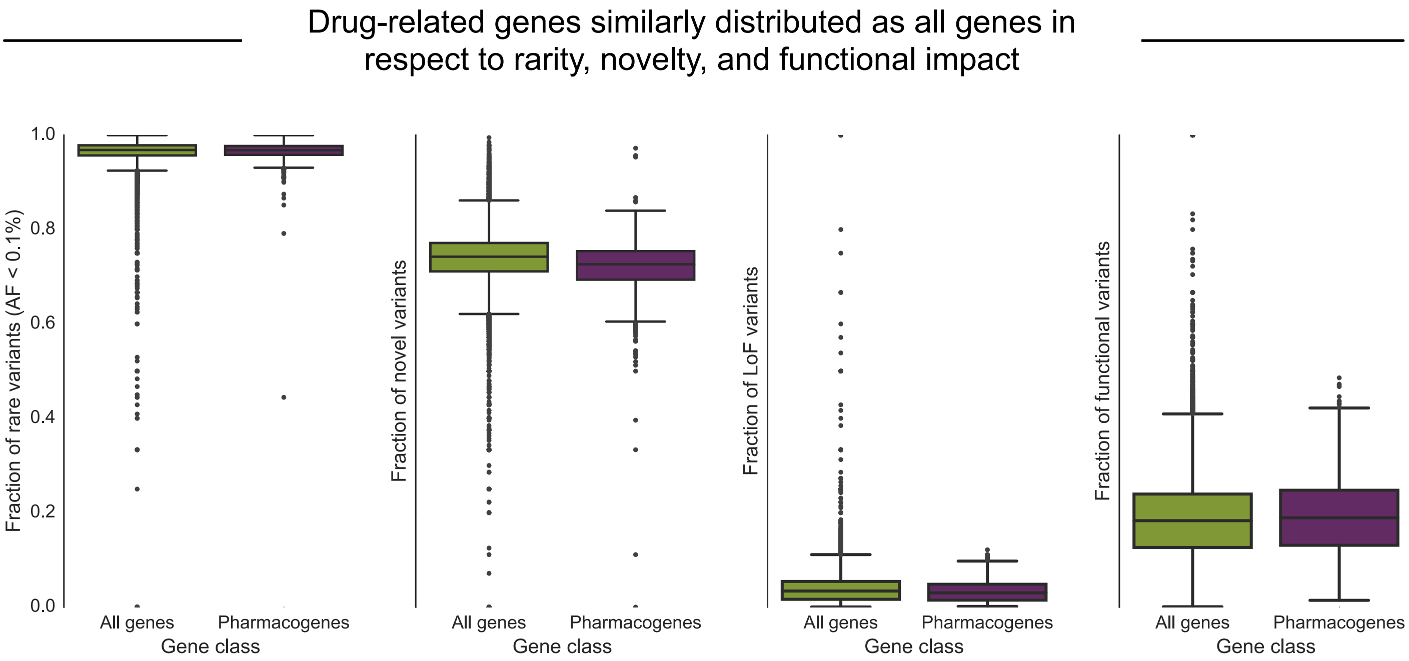

Supplementary Figure 1. Distribution of variant properties by gene in the non-synonymous subset of the ExAC collection. From left to right: fraction of variants in each gene with allele frequencies (AF) below 0.1% for all 17,758 genes compared to 806 drug-related genes (pharmacogenes); fraction of variants in gene without corresponding entries in dbSNP, thus deemed novel; fraction of variants that result in the loss of the protein product (loss-of-function, LoF) in the full data set; fraction of variants in gene that are predicted to have a functional effect (LoF or damaging as predicted by SIFT and PolyPhen).

Supplementary Figure 2

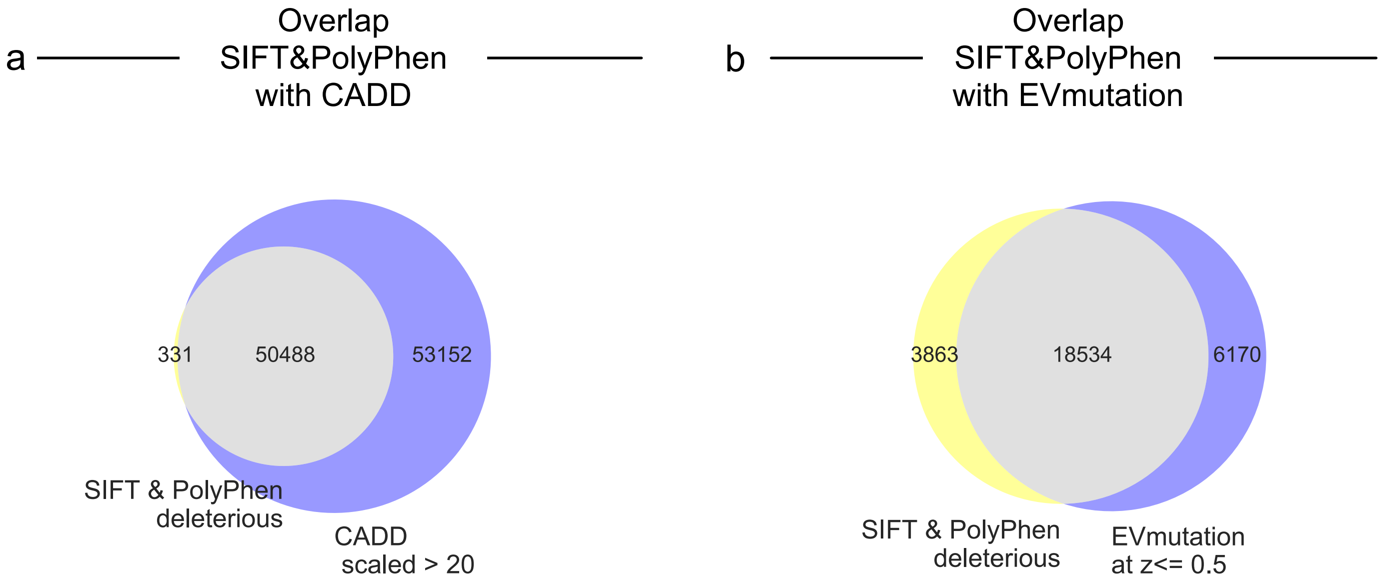

Supplementary Figure 2. Agreement between SIFT&PolyPhen consensus predictor with independent deleteriousness predictors. a) Overlap of variants classified as damaging by both SIFT and PolyPhen-2 with the set of variants classified as damaging by CADD at a scaled score threshold of 20. b) Overlap of variants classified as damaging by both SIFT and PolyPhen-2 with the set of variants classified as damaging by EVmutation at a z-score threshold of 0.5.

Supplementary Figure 3

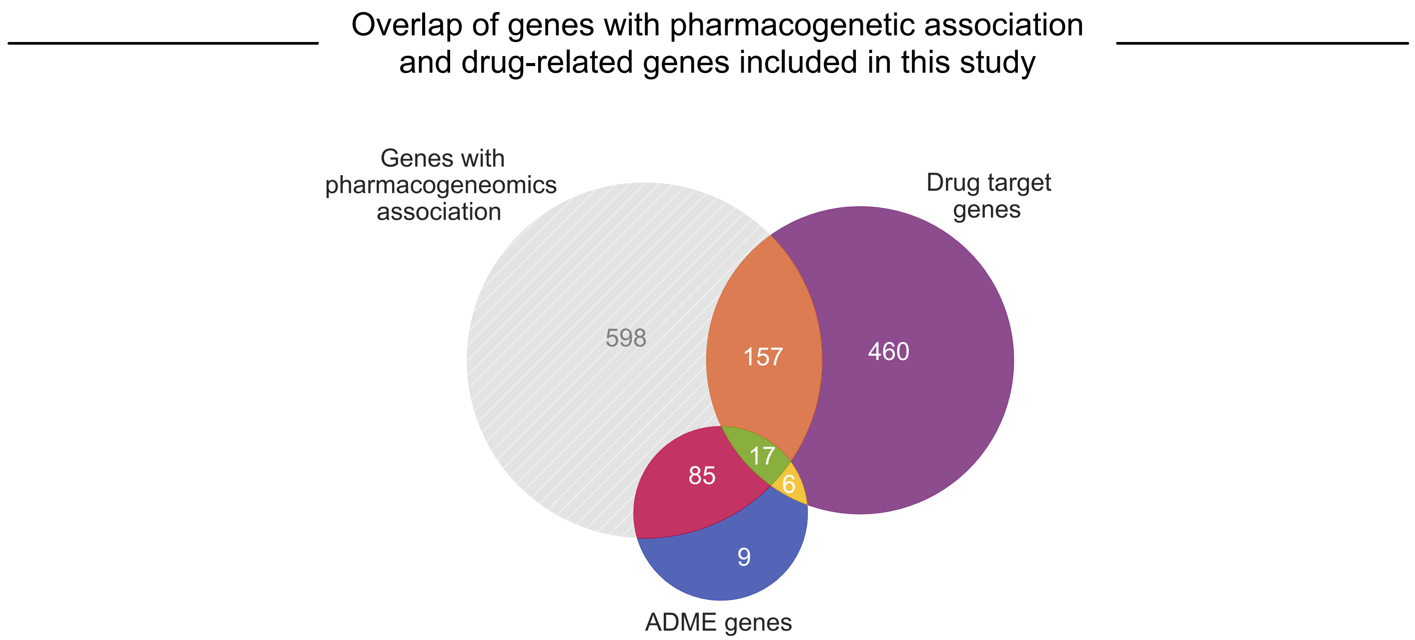

Supplementary Figure 3. Overlap between the 806 drug-related genes used in this study (drug target data collated from Drugbank 5[1] and ADME genes collated from pharmacogenomics studies[2,3]) and genes with significant pharmacogenetic association listed in pharmGKB[4].

Supplementary Figure 4

Correlation between DRP and number of drug targets

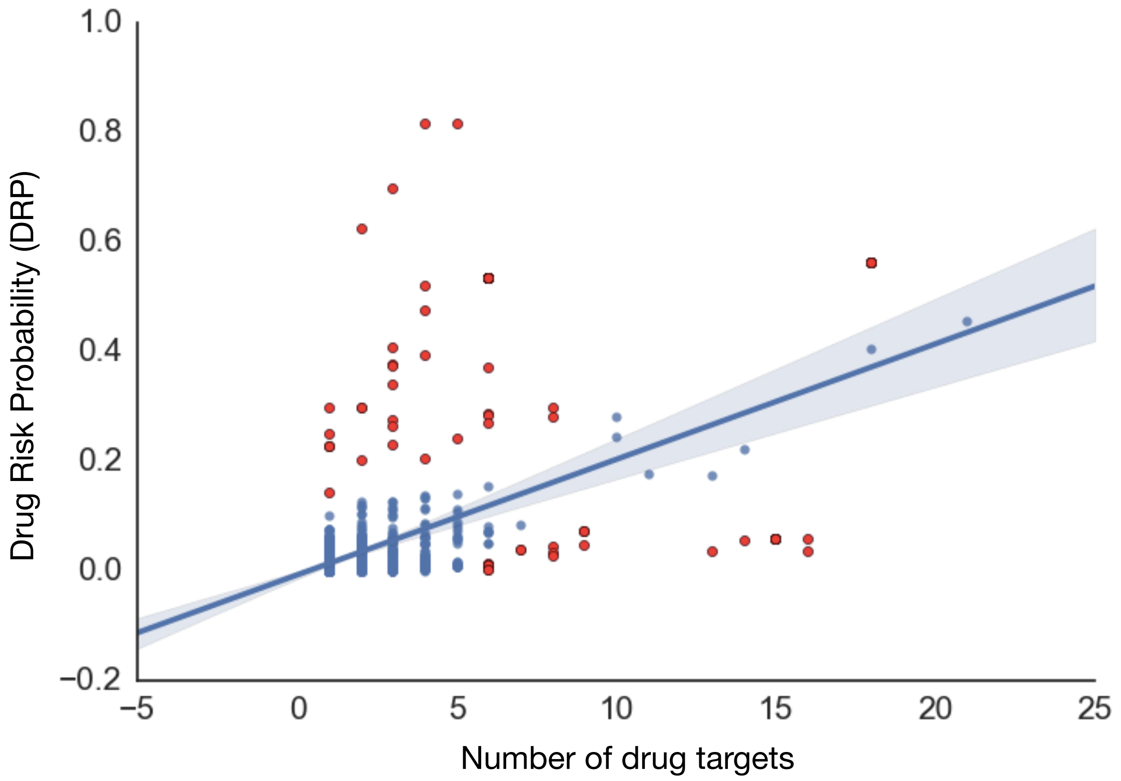

Supplementary Figure 4. Correlation between number of targets for a drug and the drug risk probability (DRP) for functional-variants in any target for a drug. Samples highlighted in red present with an RMSE > 0.01. The blue line illustrates the linear regression (with bootstrapped confidence interval in light blue).

Supplementary Figure 5

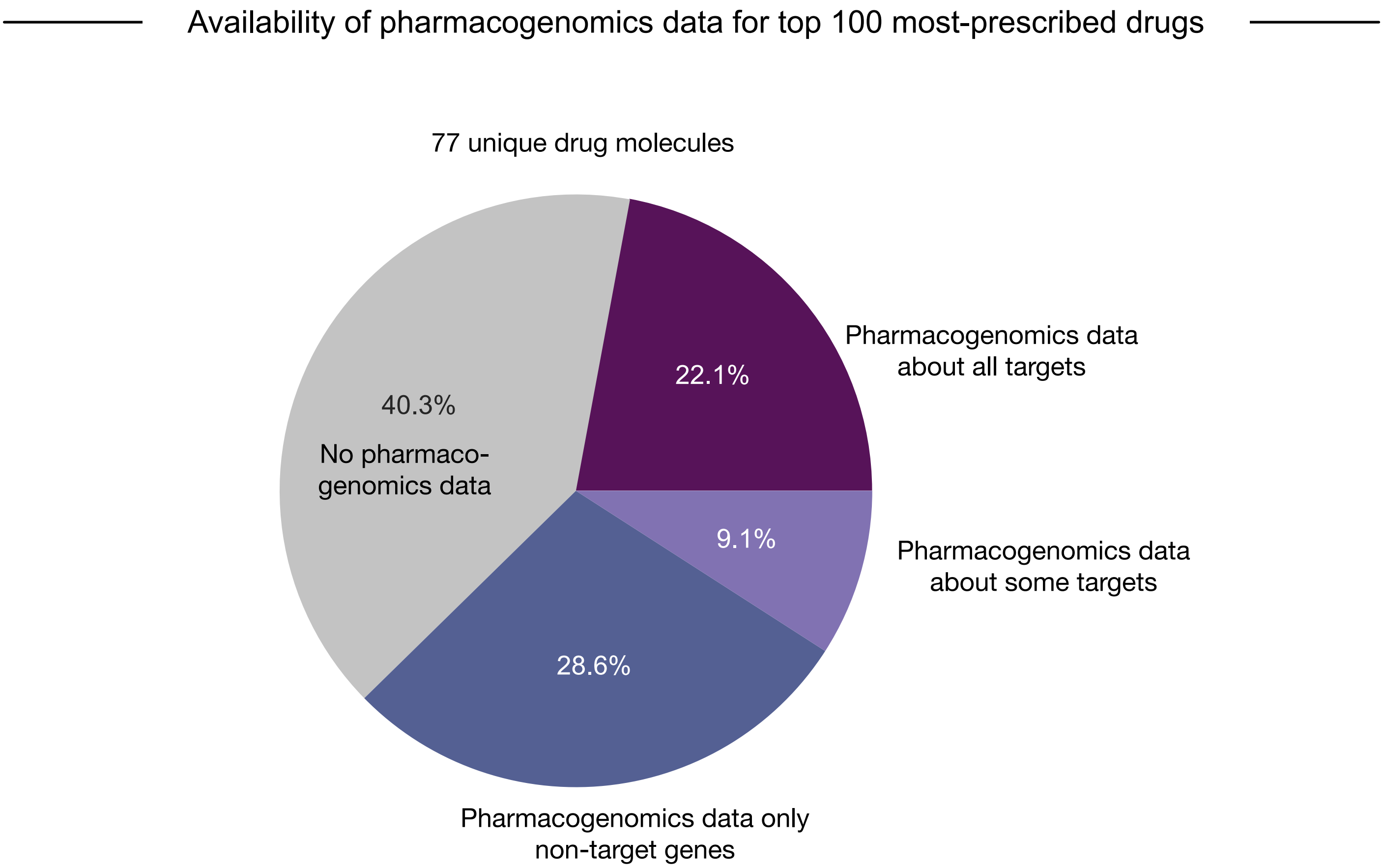

Supplementary Figure 5. Fractions of the top 100 most prescribed drugs in the US that have established pharmacogenomics data documented in the pharmacogenomics knowledge base (PharmGKB), either for genes documented to be the drug's pharmacological target in DrugBank (purple) or other genes, such as those related to drug ADME.

Supplementary Figure 6

Contact prediction for human Vitamin K epoxide reductase complex (subunit 1)

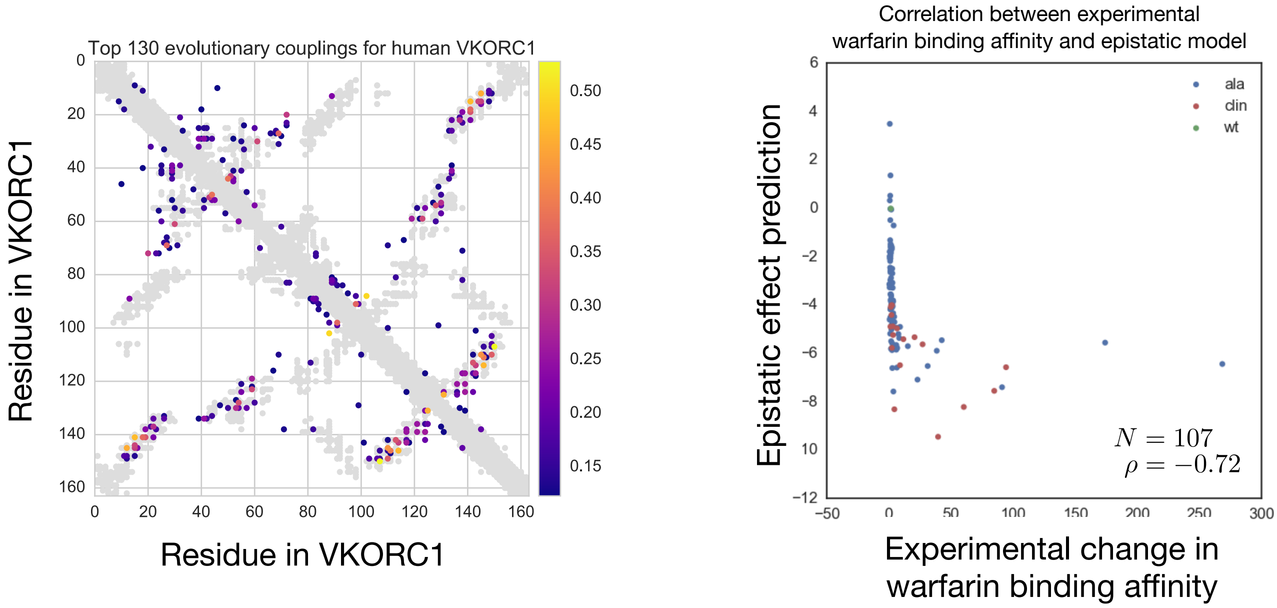

High frequency functional-variants in ExAC

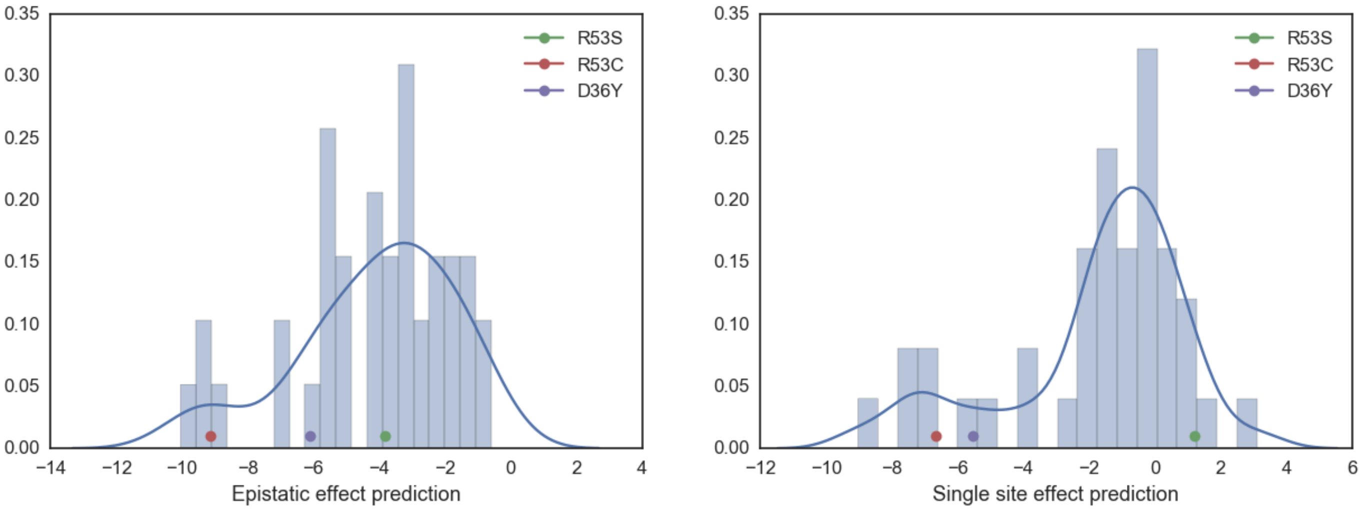

Supplementary Figure 6. Coevolution analysis of warfarin target *VKORC1*. A global maximum entropy model for human VKORC1 was built using EVfold[5] using plmc[6]. Evolutionary couplings correspond to features of the three dimensional structure of the protein (top left) and correlate with experimentally determined warfarin binding affinity for clinically observed variants (red) and alanine-scans[7,8] (blue) (Spearman rho= -0.72) (top right). Functional-variants observed in the ExAC cohort are all predicted to be less fit compared to the wild type when considering epistatic effects (bottom left panel: epistatic site model, right: single site conservation model). Positions of the three variants only observed in individual subpopulations are shown in green, red and purple.

References:

1. Law, V. *et al.* DrugBank 4.0: shedding new light on drug metabolism. *Nucleic Acids Res* **42,** D1091–7 (2014).
2. Fujikura, K., Ingelman-Sundberg, M. & Lauschke, V. M. Genetic variation in the human cytochrome P450 supergene family. *Pharmacogenetics and genomics* **25,** 584–594 (2015).
3. Kozyra, M., Ingelman-Sundberg, M. & Lauschke, V. M. Rare genetic variants in cellular transporters, metabolic enzymes, and nuclear receptors can be important determinants of interindividual differences in drug response. *Genetics in Medicine* (2016). doi:10.1038/gim.2016.33
4. Whirl-Carrillo, M. *et al.* Pharmacogenomics Knowledge for Personalized Medicine. *Clin Pharmacol Ther* **92,** 414–417 (2012).
5. Marks, D. S. *et al.* Protein 3D Structure Computed from Evolutionary Sequence Variation. *PLoS ONE* **6,** e28766–17 (2011).
6. Hopf, T. A. *et al.* Mutation effects predicted from sequence co-variation. *Nat. Biotechnol.* (2017). doi:10.1038/nbt.3769
7. Czogalla, K. J. *et al.* Warfarin and vitamin K compete for binding to Phe55 in human VKOR. *Nature Structural & Molecular Biology* **24,** 77–85 (2017).
8. Shen, G. *et al.* Warfarin traps human vitamin K epoxide reductase in an intermediate state during electron transfer. *Nature Structural & Molecular Biology* **24,** 69–76 (2017).
